# Keystone Veterinary Medical Association

**Published:** 1897-08

**Authors:** W. L. Rhoads

**Affiliations:** Secretary


### KEYSTONE VETERINARY MEDICAL ASSOCIATION.

THE final meeting for the session of 1896–97 was held in Room No. 1, third floor, N. W. corner of Broad and Filbert Sts., Tuesday evening, June 8th. The meeting was called to order by Dr. James B. Rayner, with the following members of the profession present: Drs. Drake, S. H. Johnson, A. Jolly, of Atlanta, Ga.; W. H. Hoskins, F. S. Allen, H. P. Eves, W. L. Hart, James B. Rayner, W. L. Rhoads.

Dr. W. Horace Hoskins, as Chairman of the Committee on Legislation, spoke of a bill to prevent the introduction of cattle unless accompanied by a free bill of tuberculosis, having passed both houses and now only awaits the Governor's signature to become a law. The bill reported at last meeting, which will tax all carcasses coming into the State and establish a number of meat-inspectors along our border-lines, has passed the House and is having a hard time of it. The bill to reopen registration met the fate which it merited, and did not emerge from the committee-room. It was now explained why the great decrease in the State's income had limited to a great extent the work of the State Veterinary Sanitary Board and all similar work for a time. The advisability of the veterinarian helping to procure bicycle paths through Fairmount Park, and thus encourage the driving public, was discussed.

It was then moved and seconded that the Association enter upon the discussion of "Lameness." Dr. Thomas B. Rayner had been designated as the one to open the discussion. He being absent, this duty devolved upon Dr. H. P. Eves. He spoke of the many trials and troubles we encounter in diagnosticating, seldom finding the same symptoms indicative of the same seat of trouble, and told of the benefit to be derived at times from the use of cocaine in cases where the effect might be plainly apparent while the lesions were obscure. He felt that much of our lameness was due to osteoporosis, in which theory he was upheld by nearly all of those present.

Dr. Jolly, of Atlanta, Ga, was now called upon. During the course of his remarks he spoke of a family of about thirty-five or forty horses in his vicinity, which always show a rigidity of gait, going this way probably for years, then while driving or at work they suddenly break into profuse perspiration, go down, and in a short time die. Considerable discussion followed as to the cause. Dr. Pearson spoke of one of the great show-stallions siring a number of colts which developed osteoporosis. Yet many things seem to indicate that it is infectious. Dr. Drake had seventeen cases in one stable within two years, and we find many instances of this kind regardless of location, environment, breed, or work. Dr. Pearson said it is barely possible that its cause may be a minute parasite, as in Texas fever, roup, cancer, etc., yet it is unknown in England or in Germany. The treatment suggested was lime in its varions forms. Bone-meal ground to an impalpable powder (it should first be burned, as by this process you get rid of all organic substances; it must then be specially ground).

**From New York to Nashville without change of cars.**

Dr. Eves asked why a blind horse invariably had a remarkably slick coat in the winter and a rough appearance in summer. He had often noticed it, but had never found a satisfactory explanation. Can any of our professional brothers answer, or have they, like most of those present, been going around with their eyes shut? Thus ended what has been to the members of the K. V. M. A. and their professional friends (who were enough interested in the advancement of the profession to attend the meetings which are at all times open to them) one of the most interesting and instructive sessions ever known in its history. The subjects of the half-hour talks were widely diversified, thus making it a post-graduate course, as it were, well worth the time of any progressive practitioner to attend. And the course here as elsewhere shall improve year by year. We feel that, with the exception of the great loss sustained by the death of our beloved President, Dr. John R. Hart, we have had a year of marked progression—a progression which was largely due to his indefatigable energy and efforts, and we hope to make the meetings more entertaining, more instructive, and more forcible year by year, so that each speaker or invited guest may feel, as one of our speakers expressed himself during the past session, when he said he "considered it an honor to have the privilege of addressing the K. V. M. A., the oldest local veterinary medical association in continuous existence, also the most——" but, being Secretary of the Association, modesty forbids my quoting too closely his remarks on this topic.

**Welcome all to our 34th annual convention.**

Adjourned to meet September 14, 1897.

W. L. RHOADS,
Secretary.

